# Machine Learning Algorithm for Predicting Warfarin Dose in Caribbean Hispanics Using Pharmacogenetic Data

**DOI:** 10.3389/fphar.2019.01550

**Published:** 2020-01-22

**Authors:** Abiel Roche-Lima, Adalis Roman-Santiago, Roberto Feliu-Maldonado, Jovaniel Rodriguez-Maldonado, Brenda G. Nieves-Rodriguez, Kelvin Carrasquillo-Carrion, Carla M. Ramos, Istoni da Luz Sant’Ana, Steven E. Massey, Jorge Duconge

**Affiliations:** ^1^ Center for Collaborative Research in Health Disparities (CCRHH), University of Puerto Rico Medical Sciences Campus, San Juan, Puerto Rico; ^2^ Pharmaceutical Sciences Department, School of Pharmacy, University of Puerto Rico Medical Sciences Campus, San Juan, Puerto Rico; ^3^ Department of Biology, College of Natural Sciences, University of Puerto Rico Rio Piedras Campus, San Juan, Puerto Rico; ^4^ Department of Biostatistics and Epidemiology, School of Public Health, University of Puerto Rico Medical Sciences Campus, San Juan, Puerto Rico

**Keywords:** pharmacogenetics, machine-learning, warfarin, Hispanics, prediction algorithms

## Abstract

Despite some previous examples of successful application to the field of pharmacogenomics, the utility of machine learning (ML) techniques for warfarin dose predictions in Caribbean Hispanic patients has yet to be fully evaluated. This study compares seven ML methods to predict warfarin dosing in Caribbean Hispanics. This is a secondary analysis of genetic and non-genetic clinical data from 190 cardiovascular Hispanic patients. Seven ML algorithms were applied to the data. Data was divided into 80 and 20% to be used as training and test sets. ML algorithms were trained with the training set to obtain the models. Model performance was determined by computing the corresponding mean absolute error (MAE) and % patients whose predicted optimal dose were within ±20% of the actual stabilization dose, and then compared between groups of patients with “normal” (i.e., > 21 but <49 mg/week), low (i.e., ≤21 mg/week, “sensitive”), and high (i.e., ≥49 mg/week, “resistant”) dose requirements. Random forest regression (RFR) significantly outperform all other methods, with a MAE of 4.73 mg/week and 80.56% of cases within ±20% of the actual stabilization dose. Among those with “normal” dose requirements, RFR performance is also better than the rest of models (MAE = 2.91 mg/week). In the “sensitive” group, support vector regression (SVR) shows superiority over the others with lower MAE of 4.79 mg/week. Finally, multivariate adaptive splines (MARS) shows the best performance in the resistant group (MAE = 7.22 mg/week) and 66.7% of predictions within ±20%. Models generated by using RFR, MARS, and SVR algorithms showed significantly better predictions of weekly warfarin dosing in the studied cohorts than other algorithms. Better performance of the ML models for patients with “normal,” “sensitive,” and “resistant” to warfarin were obtained when compared to other populations and previous statistical models.

## Introduction

Warfarin is one of the most used anticoagulants worldwide. However, its use tends to be challenging, due to its narrow therapeutic window and dose variability requirements among patients (Liu et al., 2015; Ma et al., 2018). Side effects may result in bleeding for patients with an overdosing or thrombosis in case of under-dosing, both related with an inadequate dosage. Consequently, patients who are under treatment need to be continuously monitored to avoid further damage. Studies have been developed in order to improve the recommended dose for warfarin patients that present side effects related to bleeding or thrombosis (Liu et al., 2015; Ma et al., 2018).

Demographic variables, genetic variants, and clinical factors are largely responsible for the broad variability of warfarin dosing among patients. Previous studies have reported that non-genetic factors such as age, height, weight, race, and drug interactions can explain around 15–20% of such inter-individual variability (Liu et al., 2015). On the other hand, genetic factors are considered critical predictors of warfarin dose requirements in various populations worldwide, particularly polymorphisms in genes encoding cytochrome P450, family 2, subfamily C, polypeptide 9 (CYP2C9) and vitamin K-epoxide reductase complex 1 (VKORC1). These two genes may individually contribute to 6–18 and 15–30%, respectively, in warfarin dose variability. However, the combination of relevant polymorphisms in both pharmacogenes accounted for approx. 30% of observed inter-patient variability in warfarin dose requirements, affecting both pharmacodynamics and pharmacokinetics of this drug (Liu et al., 2015).

To improve patient quality of life, researchers have developed predictive pharmacogenetic dosing algorithms for warfarin in multiple ethnicities (Cosgun et al., 2011; Hu et al., 2012; Liu et al., 2015; Sharabiani et al., 2015; Li et al., 2015; Ma et al., 2018). Most of the algorithms integrate demographic, clinical, and genetic variants, based on multiple linear regression (MLR) methods. Previous studies have demonstrated a prediction accuracy of around 37–55% for the patients of warfarin stable dose. In addition, machine learning (ML) algorithms in pharmacogenetic warfarin dosing have been reported (Liu et al., 2015). Some of these algorithms have been compared in racially diverse groups, however Caribbean Hispanic populations have not been included. Thus, in this study we aim to compare seven ML methods to predict stable warfarin dosing in Caribbean Hispanic patients, using genetic and non-genetic clinical data.

## Materials and Methods

### Patient Cohorts

This is a secondary analysis of genetic and clinical data collected from participants in an open-label, single-center, population-based, observational, retrospective cohort study (ClinicalTrial.gov identifier NCT01318057). Participants were recruited from the Veteran's Affairs Caribbean Healthcare System (VACHS)-affiliated anticoagulation clinic in San Juan, Puerto Rico, which serves a predominantly Caribbean Hispanic population. Participants self-reported as Caribbean Hispanic Puerto Ricans, were ≥21 years old and on a stable maintenance dose of warfarin. For the purpose of the study, a stable warfarin dose was defined as the average weekly amount of drug required to maintain stable anticoagulation levels (i.e., international normalized ratio (INR) values within therapeutic range defined as 2–3 for most indications on at least three consecutive visits). A full description of this cohort as well as detailed information on the patient's recruitment process can be found elsewhere (Duconge et al., 2016). The study was approved by the Institutional Review Boards (IRBs) of the VACHS (#00558) and the University of Puerto Rico Medical Sciences Campus (A4070109). Additional data from participants in a multicenter case–control study of Puerto Rican Hispanic patients receiving antiplatelet therapy with clopidogrel, who were recruited between January 2018 and March 2019, were also included in this secondary analysis (ClinicalTrial.gov identifier NCT03419325). This study was also IRB-approved (A4070416) by the corresponding institutional committee.

These two clinical studies were conducted according to the principles in the Declaration of Helsinki. Written informed consent was obtained from each participant prior to enrollment. Patients were divided into three major categories or classes based on their corresponding weekly warfarin dose requirements as “normal” (i.e., > 21 but <49 mg/weekly; a.k.a., intermediate dose subgroup), “sensitive” (i.e., ≤21 mg/weekly; a.k.a., low-dose subgroup), and “resistant” (i.e., ≥49 mg/weekly; a.k.a., high-dose subgroup) (Duconge et al., 2016).

### Dataset Preparation

The study dataset was prepared using information from patients of the A4070109 study cohort (N = 95), but also included data from another 95 patients in the secondary cohort (A4070416 protocol), for a total of 190 patients. Only 95 warfarin patients from the original cohort (n = 275) had full genetic, ancestry, clinical, and demographic data available to run the corresponding ML methods. Pharmacogenetic variants previously found to be associated with warfarin dose requirements in Puerto Ricans (Ramos et al., 2012; Duconge et al., 2016; Claudio-Campos et al., 2017), individual ancestry proportions, as well as clinical and demographic data from all enrolled patients were considered in the corresponding analyses. The primary cohort, which corresponds to patients on warfarin, included 40 “normal,” 38 “sensitive,” and 17 “resistant” cases. All cases from the secondary cohort were assigned to the “normal” weekly warfarin dose category. Their doses were imputed as random values within ±20% of the average dose level in the “normal” subgroup of the primary cohort.

To develop and evaluate the models, the data was separated as approximately 80% for the training set (N of training = 154) and about 20% for the testing set (N of testing = 36). The training set had an imbalanced distribution for the number of “normal” cases (“normal” = 111), *versus* “sensitives” and “resistant” (“sensitive” = 30, “resistant” = 13). Then, a randomized oversampling technique was used to balance the training dataset in order to develop the models (Ling and Li, 1998).

### Genotyping and Ancestry Estimations

All DNA specimens from participants were tested following manufacturer's instructions. A full description of genotyping methods can be found elsewhere (www.illumina.com/genotyping). Briefly, the Infinium™ Human OmniExpress-24 v1.2 BeadChip by Illumina, which provides a broad coverage of relevant markers for genome-wide association studies (GWAS), was used to perform the genetic testing of 95 warfarin patient from the A4070109 study cohort in iScan^®^ system (Illumina, San Diego, CA). Additionally, the Infinium™ Multi-Ethnic Global AMR/AFR BeadChip was used in the A4070416 cohort of patients on clopidogrel. Genotypes at relevant loci (i.e., *FMO2* c.107A > G, p.D36G, rs2020870 in chromosome 1; *ABCB1* c.1000-44G > A, rs10276036 in chromosome 7; *SLCO1B3* c.1833G > A, G611 =, rs3764006 in chromosome 12; *CYP2C9*, rs1856908 and CYP2C9*2 c.430C > T, p.R144C, rs1799853 in chromosome 10; *VKORC1* c.1173C > T, rs9934438 in chromosome 16; CYP4F2*3 c.1297G > A, p.V433M, rs2108622 in chromosome 19; NQO1*2 c.4559C > T, p.P187S, rs1800566 in chromosome 16) were then retrieved from the corresponding Variant Call Format (VCF) files.

Individual proportions of each ancestry component in the study population were estimated by ADMIXTURE software (Alexander et al., 2009), with the corresponding parental references for the Native American (NAT), European (EUR), and African (AFR) contributions taken from the 1,000 Genomes Project (Auton et al., 2015). To this purpose, data from Iberian populations in Spain (IBS) and Yoruba population in Ibadan, Nigeria (YRI) were used to properly represent EUR and AFR ancestries in the analysis, respectively.

### Machine Learning Algorithms

Seven ML algorithms were selected for generating the models and testing them using the data from the two Caribbean Hispanic cohorts. These algorithms were multivariate adaptive splines (MARS) (Klein et al., 2009), artificial neural networks (ANN) (Grossi et al., 2013), random forest regression (RFR) (Cosgun et al., 2011), support vector regression (SVR) (Suykens and Vandewalle, 1999), K-nearest neighbor for K from 1 to 3 (i.e., iBK1, iBK2, iBK3, respectively) (Aha et al., 1991), recursive partitioning (RPART) (Breiman, 1984), and reduces error tree classifier (REPT) (Mohamed et al., 2012). Weka—ML in Java software was used to both train the ML algorithms and obtain the predictive models, as well as evaluate and compare the models (Frank et al., 2016). For each ML algorithm tested, the model with the best predictability was chosen regardless of the number of added variants.

To evaluate and compare the model's predictability, we primarily computed the mean absolute error (MAE) and the percentage (%) of patients whose predicted warfarin dosage values were within 20% of the actual stable dosage found in the available data (Duconge et al., 2016). This 20% value represents a difference of 7 mg/week relative to the standard starting dose of 35 mg/week, a difference that clinicians define as clinically relevant. The MAE is the average of the absolute difference between two continuous values, in this case the actual and the predicted dose values. Both metrics (i.e., MAE and percentage within 20%) were compared among the ML models independently and after dividing patients into the above-mentioned three categories based on their warfarin dose requirements (i.e., “normal,” “sensitive,” and “resistant”).

### Statistical Analyses

All comparisons of mean values between training and test datasets were performed by using a two-sided unpaired t-test (Hsu, 1938) for continuous variables (e.g. warfarin dose, weight, ancestry estimates, etc.) and a proportion-test (Wilson, 1927) for frequencies or dichotomous variables (e.g. diplotypes, conditions, co-medications, etc.).

## Results

### Basic Characteristics of the Study Cohort

Clinical and demographic variables of interest are summarized in **Table 1** for the 190 patients included in this study (i.e., 154 assigned to a training set and another 36 in the test set). **Table 1** also presents their corresponding ancestry proportions. Furthermore, diplotypes at each genetic locus of interest in this study are also shown in **Table 2**.

**Table 1:** Relevant characteristics of the Caribbean Hispanic patients included in this study.

Variables	Groups		*p*-value	Total cohort (n = 190)
	Training set (n = 154)	Test set (n = 36)		
**Warfarin dose** (mg/week), mean (SD)	32.59 (8.99)	32.84 (7.68)	0.8627	32.64 (8.74)
**Weight** (kg), mean (SD)	81.27 (18.67)	81.42 (16.12)	0.9612	81.29 (18.17)
**Height** (cm), mean (SD)	167.45 (8.73)	168.94 (8.36)	0.3440	167.74 (8.66)
**Ancestry proportions – mean (SD)**				
**NAT**	0.11 (0.03)	0.11 (0.03)	0.9419	0.12 (0.05)
**EUR**	0.68 (0.13)	0.70 (0.10)	0.4789	0.68 (0.14)
**AFR**	0.20 (0.14)	0.19 (0.10)	0.5026	0.20 (0.13)
**Population by age (%)**				
**≥50 years-old**	151 (98.05)	33 (91.67)	0.1915	184 (96.84)
**<50 years-old**	3 (1.95)	3 (8.33)	0.1915	6 (3.16)
**Conditions (%)**				
**DVT**	12 (7.79)	6 (16.67)	0.1897	18 (9.47)
**PE**	4 (2.60)	4 (11.11)	0.1273	8 (4.21)
**AF**	50 (32.47)	16 (44.44)	0.1995	66 (34.74)
**VR**	5 (3.25)	2 (5.56)	0.5788	7 (3.68)
**Stroke**	9 (5.84)	5 (13.89)	0.1975	14 (7.37)
**DM2**	73 (47.4)	18 (50.0)	0.7826	91 (47.89)
**CHF**	9 (5.84)	5 (13.89)	0.1975	14 (7.37)
**Smokers**	15 (9.74)	5 (13.89)	0.5146	20 (10.53)
Others*	101 (65.58)	21 (58.33)	0.4331	122 (64.21)
**Co-medications (%)**				
**Aspirin**	56 (36.36)	10 (27.78)	0.3175	66 (34.74)
**Statins**	101 (65.58)	21 (58.33)	0.4331	122 (64.21)
**Azoles**	5 (3.25)	1 (2.78)	0.8813	6 (3.16)
**Clopidogrel^∦^**	79 (51.3)	16 (44.4)	0.8700	95 (50)

Mean refers to arithmetic mean. NAT, Native Americans; AFR, Africans; EUR, Europeans; DVT, Deep Vein Thrombosis; PE, Pulmonary Embolism; AF, Atrial Fibrillation; VR, Valve Replacement; DM2, Type-2 Diabetes Mellitus; CHF, Congestive Heart Failure. *Others means any other diagnosis of cardiovascular conditions (e.g., acute coronary syndrome, peripheral artery disease, chronic hypertension, etc.). ^*∦*^clopidogrel doses of 75mg/daily.

**Table 2:** Frequency distributions of relevant genotypes in the Caribbean Hispanic patients included in this study.

Genotypes	Groups		p-value	Total cohort (n = 190)
	Training set (n = 154)	Test set (n = 36)		
**at locus 1, Chr1: *FMO2* c.107A > G, p.D36G, rs2020870 (%)**				
**A/A**	84 (54.56)	23 (63.89)	0.3072	107 (56.32)
**A/G**	48 (31.17)	12 (33.33)	0.8068	60 (31.58)
**G/G**	20 (12.99)	1 (2.78)	0.0010	21 (11.05)
**Unknown** ^#^	2 (1.30)	0	–	2 (1.05)
**at locus 2, Chr7: *ABCB1* c.1000-44G > A, rs10276036 (%)**				
**G/G**	81 (52.60)	28 (77.78)	0.9986	109 (57.37)
**G/A**	59 (38.31)	8 (22.22)	0.9748	67 (35.26)
**A/A**	13 (8.44)	0	–	13 (6.84)
**Unknown^#^**	1 (0.65)	0	–	1 (0.53)
**at locus 3, Chr12: *SLCO1B3* c.1833G > A, G611 =, rs3764006 (%)**				
**G/G**	82 (53.25)	27 (75)	0.9941	109 (57.37)
**G/A**	51 (33.12)	8 (22.2)	0.1781	59 (31.05)
**A/A**	20 (12.99)	1 (2.78)	0.9109	21 (11.05)
**Unknown^#^**	1 (0.65)	0	–	1 (0.53)
**at locus 4, Chr10: *CYP2C9*, rs1856908 (%)**				
**A/A**	94 (61.04)	26 (72.2)	0.1955	120 (63.16)
**A/G**	47 (30.52)	9 (25.0)	0.5043	56 (29.47)
**G/G**	13 (8.44)	1 (2.78)	0.1166	14 (7.37)
at locus 5, Chr10: CYP2C9*2 c.430C > T, p.R144C, rs1799853 (%)				
**C/C**	129 (83.7)	30 (83.3)	0.9961	159 (83.5)
**C/T**	24 (15.6)	6 (16.6)	0.8102	30 (15.8)
**T/T**	1 (0.65)	0	–	1 (0.77)
**at locus 6, Chr16: *VKORC1* c.1173C > T, rs9934438^§^ (%)**				
**C/C**	75 (48.70)	12 (33.33)	0.1388	87 (45.79)
**C/T**	62 (40.26)	17 (47.22)	0.5651	79 (41.58)
**T/T**	17 (11.04)	7 (19.44)	0.2765	24 (12.63)
at locus 7, Chr19: CYP4F2*3 c.1297G > A, p.V433M, rs2108622 (%)				
**C/C**	69 (44.80)	25 (69.44)	0.0133	94 (49.47)
**C/T**	70 (45.45)	9 (25.0)	0.0399	79 (41.58)
**T/T**	15 (9.74)	2 (5.56)	0.6400	17 (8.95)
at locus 8, Chr16: NQO1*2 c.4559C > T, p.P187S, rs1800566 (%)				
**C/C**	77 (50.0)	23 (63.39)	0.1878	100 (52.63)
**C/T**	60 (38.96)	11 (30.56)	0.4549	71 (37.37)
**T/T**	17 (11.04)	2 (5.56)	0.4973	19 (10.00)

**^#^**unknown genotype indicates a missing or non-calling at this particular locus. ^§^The *VKORC1*c.-1639G > A (rs9923231) and c.1173C > T (rs9934438) SNPs are in near complete linkage disequilibrium in individuals of European, Asian and African descent (Cavallari and Momary, 2013).

Among these 190 patients, 96.8% were aged 50 years or older. Their average warfarin dose was 32.6 mg/week with a standard deviation of 8.74. A total of 122 patients were using statins to lower their cholesterol levels. Of note is the relatively low prevalence of CYP2C9*2 carriers in the study cohorts, with only 16% of single and double carriers combined (minor allele frequency (MAF) = 0.08). About 50–60% are homozygous for the major alleles (i.e., wild-types) across all other pharmacogenetic loci tested in this study; whereas, the percentage of heterozygous at each of these polymorphic sites ranged from 29.5 to 41.6%. Accordingly, a relatively low number of patients were homozygous for the variant allele and just a few of them had unknown genotypes at these loci and, therefore, were excluded from subsequent analyses.

The p-values in **Tables 1** and **2** correspond to the statistical comparisons of relevant characteristics between the training and test sets. Overall, no significant differences between both sets were found with regard to their pharmacogenetics, ancestry, clinical, and demographic variables. Accordingly, these two sets of data are comparable to each other as they were matched by all these relevant variables. Likewise, all genotypes and allelic frequencies of the genetic markers included in this study were in Hardy-Weinberg (HW) equilibrium, as no significant departure from HW assumptions were found.

### Overall Comparison of Predictive Algorithms

As can be seen in **Table 3**, with a MAE of 4.73 mg/week and a percentage within 20% of 80.6, RFR was significantly better in predictability than the other developed models. Indeed, all these other models fell short in their performances to predict optimal doses (i.e., MAEs of 6.15–9.87 mg/week and predictions of 47.22–72.22% of ideal doses) when compared to RFR. The MAE values lie within the 6.00–7.00 mg/weekly range in five of these algorithms (i.e., SVR, RPART, iBK1, iBK2, and iBK3). Notably, REPT, ANN, and MARS had the worst performances as suggested by their corresponding MAE values and % predictions within 20% of the ideal doses (8.52–9.87 mg/week and 47.2–58.3%, respectively). Interestingly, the combination of novel and common variants across the pharmacogenes of interest improved model's predictability in all but SVR and REPT algorithms, with −5 and −18% of ideal dose predictions (i.e., within 20%) after adding common variants of previously demonstrated clinical relevance.

**Table 3:** Mean absolute error (MAE) and percentage within 20% of actual dose in the overall test set of the Caribbean Hispanic cohort.

Models	MAE (mg/week)	Within 20%
***RPART***	6.27 (4.16-8.38)	72.22
***MARS***	8.52 (5.92-11.12)	55.56
***RFR***	4.73 (3.24-6.21)	80.56
***ANN***	9.73 (6.53-12.93)	58.33
***SVR^#^***	6.86 (4.75-8.97)	61.11
***iBK1***	6.78 (4.41-9.15)	66.67
***iBK2***	6.30 (3.88-8.71)	69.44
***iBK3***	6.15 (3.72-8.57)	72.22
***REPT**^#^*	9.87 (6.62-13.12)	47.22

***^#^***best prediction model does not include common variants in *VKORC1* (rs9923231), *CYP2C9* (rs1799853), *CYP4F2* (rs2108622) and *NQO1* (rs1800566). Data are expressed as mean (95% CI) or percentage. MAE, mean absolute error; multivariate adaptive regression splines (MARS), artificial neural networks (ANN), random forest regression (RFR), support vector regression (SVR), K-nearest neighbor for K from 1 to 3 (iBK1, iBK2, iBK3), recursive partitioning (RPART) and reduces error pruning tree classifier (REPT).

### Comparison of Predictive Algorithms Within Warfarin Dose Range

In general, these ML algorithms performed better in the subgroup of patients with normal dosing requirements. Nonetheless, the RFR algorithm was again the best in terms of MAE (2.91 mg/week) and within 20% (100%) when compared to the other methods. In the subgroup with low dose requirements (sensitives), SVR and RFR significantly outperformed all other methods in MAE (4.79–7.17 mg/week, respectively) and within 20% (75.00–50.00%, respectively). For the resistant patients, MARS was the best algorithm in both MAE (7.22 mg/week) and % values within 20% (66.67), though iBK2 and iBK3 also showed good results (7.58 mg/week and 66.67%). Overall, the models generated for the subgroup with normal warfarin dose requirements performed better than those used to predict dosing among sensitives and resistant patients (**Table 4**). Strikingly, when models included both common and novel variants combined their predictability improved in general, except for the sensitive subgroup where performances were as bad as −67% of ideal dose predictions (i.e., within 20%) in comparison to the models excluding the common variants. In the resistant subgroup, only MARS had a worse performance (−50%) after adding the common variants (**Supplementary Material S1**).

**Table 4:** Mean absolute error and mean percentage within 20% of actual dose stratified by therapeutic warfarin dose requirements (i.e., sensitives, resistant and normal) in the test set of the Caribbean Hispanic cohort.

Models	Normal		Sensitive		Resistant	
	MAE (mg/week)	Within 20%	MAE (mg/week)	Within 20%	MAE (mg/week)	Within 20%
**RPART**	4.17 (2.60-5.73)	88.00	9.83 (3.53-16.12)	37.50	14.28 (7.19-21.37)	33.33
**MARS**	6.98 (4.44-9.52)	68.00	11.44 (6.55-16.33)***^#^***	25.00	7.22 (2.11-12.33)***^#^***	66.67
**RFR**	2.91(2.18-3.64)***^#^***	100.00	7.17 (3.48-10.86)***^#^***	50.00	13.45 (7.41-19.48)	33.33
**ANN**	8.03 (5.01-11.05)	68.00	10.32 (1.49-19.15)	50.00	16.75 (10.87-22.63)***^#^***	0.00
**SVR**	5.67 (3.82-7.53)***^#^***	68.00	4.79 (1.21-8.36)	75.00	19.44 (15.83-23.05)	0.00
**iBK1**	3.62 (2.01-5.24)	88.00	12.81 (6.15-19.48)***^#^***	25.00	12.83 (2.48-23.18)	33.33
**iBK2**	3.75 (1.94-5.55)	88.00	9.33 (2.17-16.49)***^#^***	37.50	7.58 (2.33-17.50)	66.67
**iBK3**	3.83 (2.01-5.64)	88.00	9.33 (2.17-16.49)***^#^***	37.50	7.58 (2.33-17.50)	66.67
**REPT**	6.89 (4.68-9.09)***^#^***	56.00	12.30 (2.60-22.01)***^#^***	37.50	15.08 (2.67-27.50)	66.67

***^#^***best prediction model does not include common variants in *VKORC1* (rs9923231), *CYP2C9* (rs1799853), *CYP4F2* (rs2108622) and *NQO1* (rs1800566). Data are expressed as mean (95% CI) or percentage. MAE: mean absolute error; multivariate adaptive regression splines (MARS), artificial neural networks (ANN), random forest regression (RFR), support vector regression (SVR), K-nearest neighbor for K from 1 to 3 (iBK1, iBK2, iBK3), recursive partitioning (RPART) and reduces error pruning tree classifier (REPT).

## Discussion

Overall, we found different performances of the nine ML-based algorithms that were used to predict warfarin dosing in the Caribbean Hispanic population (**Table 3**). When all the cases were considered, the RFR algorithm achieved the best performance. However, RFR, SVR, and MARS algorithms had the best performance when the patients were grouped by dose range as “normal,” “sensitive,” “resistant,” respectively. There is no obvious explanation or a given reason why these specific models performed better than the others. It is because algorithms derived from ML techniques are based on choosing the best model as they learn from data (Brownlee, 2019). Therefore, it seems to be population dependent.

The model with best predictability was chosen for each of the ML-based algorithms tested, regardless of the number of added variants. However, we tried to keep the models as simple as possible (i.e., minimum number of parameters or variables) while preserving a reasonably great explanatory predictive power. Since models with low parsimony will likely be useless for predicting other datasets, we chose the models with the right balance between parsimony and goodness of fit.

### Comparison to Previous Algorithms for Dose Predictions in Other Populations

The performance of similar ML methods applied to warfarin dose predictions have shown different results in a previous study (Liu et al., 2015). Of note is that no significant differences in overall performances of various ML-based algorithms were reported by others when used as a prediction tool for stable warfarin dose estimations in a multi-ethnic cohort. However, differences in model accuracy were indeed found after stratifying data by ethnicity (i.e., White *vs.* Asians *vs.* Blacks) or dose range subgroups (i.e., high *vs.* intermediate *vs.* low) (Liu et al., 2015; Ma et al., 2018). We have obtained better MAEs than this previous report for the analyses of data from all cases in most of the tested ML methods (e.g. RFR, SVR, RPART, iBK1). When datasets from Liu et al. (2015) and our study were compared, the best result for all cases was obtained with the use of the RFR technique in our dataset of Caribbean Hispanics (i.e. MAE = 4.73 mg/week and 80.56% cases within ±20% of ideal doses). We reason that these observed differences in performance may have arisen as a consequence of the unique genetic backgrounds, clinical characteristics of our study cohort (Caribbean Hispanics), and special attributes of the available dataset (e.g., genetic markers for resistance, ancestry metrics). Accordingly, such findings may be attributed to differences in the characteristics of participants from both studies and the fact that the previous one was conducted in a more heterogeneous cohort of individuals from the International Warfarin Pharmacogenetics Consortium (IWPC), without a proper representation of Caribbean Hispanics (Liu et al., 2015). It is important to mention that the IWPC cohort comprised a mixed sample from different countries, regions, and clinical sites that could lead to misclassification and large genetic variability. Finally, it may also be related to the unequal sample sizes between both studies.

Similarly, after grouping patients by dose requirements (i.e. “normal,” “sensitive,” and “resistant” to warfarin, **Table 4**), the ML prediction models in our study performed better than those in the published report (Liu et al., 2015). In those labeled as “normal,” our best model (RFR) yielded a MAE = 2.91 mg/week to outperform the 5.53 mg/week from the study by Liu et al. (2015). For “sensitive” patients, the SVR is our best model with a MAE = 4.79 mg/week that resulted more accurate for predictions in this subgroup than the value of 8.68 mg/week from the previous report (Liu et al., 2015). Finally, those warfarin patients classified as “resistant” had a MAE = 7.22 mg/week with our best-performance model in this subgroup (MARS), which is far better than the reported 15.24 mg/week in the previous study by Liu et al. (2015).

As expected, our results indicate that both the MAEs and mean percentages within 20% of all algorithms under consideration differed across the dose range categories (i.e., “normal,” “sensitive,” and “resistant”), with best performance and accuracy (i.e., lower MAE and higher mean percentage within 20%) achieved in the “normal” dose group and “resistant” showing the worst predictions. In fact, the largest difference in the MAE and percentage within 20% were observed between “normal” and “resistant” subgroups. However, better predictors do not really translate into a real clinical utility to this “normal” subgroup as patients in this class are least likely to benefit from pharmacogenetics (Klein et al., 2009). Consequently, benefits are mainly for those at the extreme dose requirements. “Resistant” demonstrated to have the highest variability in warfarin dose requirements among patients at any dosing range, suggesting that either current ML-based methods are not yet robust enough to optimally predict dosing in patients with a resistant phenotype or the lack of information from all predictors of resistance to warfarin in the model. Since ML techniques learn from existing data, the insufficient number of “resistant” cases in available dataset and, therefore, the limited amount of relevant data that can inform the model, may in part explain the poorer performance at this dose range. Accordingly, efforts should be made in order to enhance the representation of this sub-group in future assessments.

### Comparison to Previous Algorithms for Dose Predictions in Caribbean Hispanics

Our group has earlier published three previously developed pharmacogenetic algorithms to predict optimal warfarin dosing in Caribbean Hispanics of mostly Puerto Rican origin, which included ethno-specific alleles and adjustments by admixture measures in the derivation cohort (Ramos et al., 2012; Duconge et al., 2016; Claudio-Campos et al., 2017). All these models were based on multivariate linear regression analyses. Overall, they showed a good predictability in our patients to outperform prior genotype-guided algorithms derived from populations other than Hispanics. When using these regression pharmacogenetic models, up to 46% of their predictions in high risk individuals resulted in ideal doses (i.e., % of predictions within ±20% of the actual patient's stabilization dose) with MAE values that sit slightly over 5 mg/week. However, some ML-based models developed in this survey by using RFR, MARS, and SVR approaches showed even better results in predicting optimal warfarin doses in the study cohort as compared to the previously published regression methods. Particularly, the overall performance of the RFR model was better than published algorithms, as suggested by a MAE of less than 5 mg/week and 80.6% of ideal dose predictions. Among those at the highest risk of adverse events, both SVR and RFR showed superiority over the previously published regression algorithms with higher percentages of ideal dose predictions (i.e., 50 and 62%, respectively). Notably, the ML-based methods (RFR, SVR) performed better than previous linear regression models in both high- and low-dose subgroups (i.e., resistant and sensitives). Therefore, this analysis reflects the potential of ML techniques for predictions at extreme dose levels given their capabilities to assess patient characteristics under extreme dosing requirements. A possible explanation for this observed superiority of ML models over the conventional algorithms is given by the fact that these applications of artificial intelligence (AI) provides systems the ability to automatically learn and improve predictability from experience (i.e., available data).

### The Missing Links for Global Pharmacogenomics

Most of the existing pharmacogenetic-driven algorithms such as the one developed by the IWPC project have been derived from findings in individuals of mostly European descent, and therefore they often include variants commonly found in white people only. Multiple ethno-specific variants occurring across warfarin-related pharmacogenes are generally overlooked and, consequently, the utility of existing prediction models is limited in patients with mixed ancestry. Healthcare disparities could be exacerbated when such models are not suitable to populations with ethno-geographic particularities.

White people of European ancestry make up the largest percent of participants in pharmacogenomic (PGx) studies, despite the fact that they only represent a fraction of the world's population. Furthermore, clinically relevant findings from such studies with Europeans do not generalize well to other ethnic groups. This overwhelming whiteness of pharmacogenetics research is holding back the new paradigm of precision medicine. One of the greatest promises of the Precision Medicine initiative is the opportunity to develop treatment plans that are tailored to an individual's genetic risk profile. Therefore, if individuals from underrepresented populations are not involved in these investigations, they will not benefit from the advances. Indeed, there is a paucity of data from studies recruiting minority, more diverse or admixed populations like Caribbean Hispanics who reside in Puerto Rico. Unfortunately, individuals from these populations are often excluded or marginally represented in these studies and this lack of representation tends to exacerbate existing healthcare disparities. It's adding to the long-standing problem of minorities being excluded from medical research, which preclude any opportunity to make them equitable.

MLR analysis routinely used to derive pharmacogenetic models is data driven and hence population dependent. There is promising research indicating that mathematical models other than linear regression may yield more predictive algorithms (Cosgun et al., 2011; Hu et al., 2012; Liu et al., 2015; Sharabiani et al., 2015; Duconge and Ruaño, 2018; Ma et al., 2018). AI, and particularly the use of ML techniques, offers new avenues in the prediction of clinical outcomes (e.g., warfarin dose requirements) by accounting for relevant gene–drug interactions. Failure to account for ethno-specific genotypes and a better use of available predictive tools (e.g., ML) has raised some concerns about expected benefits of genotyping patients to guide pharmacotherapies and improve clinical outcomes, leading to a lack of full endorsement by medical organizations and payers. The more complete the PGx characterization and the more learned the prediction models, the larger the benefit.

This study has some limitations. Firstly, some data were retrospectively collected and, therefore, we were unable to control for such data variability and potential confounders. Given a relatively lesser representation of cases at the extreme dose levels with respect to those in the “normal” range, a potential bias may arise in the comparison after subgrouping by dosing requirements. For the purpose of the analyses in this paper, we considered “normal” responders as those without any obvious or given reason to make adjustments in their standard initial warfarin dose (i.e., 35 mg/week; range: 21–49 mg/weekly). Theoretically speaking, we reasoned that those from the clopidogrel study can be considered as “normal” because of a lack of any obvious reason for starting these patients with a different dosing (e.g., frail elderly, high risk of bleeding/thrombosis, etc.) had they been treated with warfarin. However, this assumption should be observed with caution and, hence, is another study limitation. Finally, our findings need further validation in a larger replication cohort before making any statement about the superiority of some of these algorithms over the others.

The metrics to assess the performance of algorithms developed in other studies are not comparable to those used in this study, whose methodology is mainly based on an early work by Liu and coworkers (Liu et al., 2015). Unlike previous reports, in this study we have included genetic markers for both sensitivity and resistance phenotypes, and admixture/ancestry estimates as critical covariates in model development (Klein et al., 2009; Ramos et al., 2012; Liu et al., 2015). Moreover, relevant data from a highly diverse admixed population of Caribbean Hispanics is used for the first-time to perform an ML prediction modeling of a pharmacogenetic trait. MAEs and percentage of predictions within ±20% revealed that models generated by using RFR, MARS, and SVR ML algorithms showed significantly better predictions of warfarin dosing in our cohort of participants than other algorithms. Better performance of the ML models for patients with “normal,” “sensitive,” and “resistant” to warfarin were obtained in our study as compared to other populations and previous statistics models.

## Data Availability Statement

The datasets generated for this study can be found in the dbGaP, Study Accession: phs001496.v1.p1, https://www.ncbi.nlm.nih.gov/projects/gap/cgi-bin/study.cgi?study_id=phs001496.v1.p1.

## Ethics Statement

The studies involving human participants were reviewed and approved by Human Research Subjects Protection Office (HRSPO) affiliated to the University of Puerto Rico Medical Sciences Campus, (IORG000223; Federal-wise Assurance #FWA00005561). The patients/participants provided their written informed consent to participate in this study.

## Author Contributions

AR-L provided the infrastructure, supervised the analysis of the data using bioinformatics tools, and participated in the writing of the manuscript. AR-S and RF-M both performed most of the data analyses and drafted the original version of the manuscript. They also worked with the corresponding author in data collection and assembly. JR-M, BN, and KC-C performed part of the data analysis. CR and IS'A performed the statistics of this study and contributed to the manuscript preparation. SM contributed in the preparation of the manuscript. JD is the principal investigator, responsible of the study coordination, and contributed to the elaboration of the manuscript.

## Funding

This work was supported by CCRHD-RCMI grant # 2U54 MD007600-31 from the National Institute on Minority Health and Health Disparities (NIMHD) of the National Institutes of Health. Part of this research was also supported by SC1 grant # HL123911 from the National Heart, Lung and Blood Institute (NHLBI) and the MBRS SCORE Program of the National Institute of General Medical Sciences (NIGMS). Disclaimer: The contents of this manuscript do not represent the views of the Veterans Affairs Caribbean Healthcare System, the Department of Veterans Affairs, the National Institutes of Health, or the United States Government. No funded writing assistance was utilized in the production of this manuscript.

## Conflict of Interest

JD held a without compensation (WOC) employment status with the Pharmacy Service, VA Caribbean Healthcare Systems (VACHS), in San Juan, Puerto Rico, at the time of conducting the study.

The remaining authors declare that the research was conducted in the absence of any commercial or financial relationships that could be construed as a potential conflict of interest.

The reviewer MB declared a past co-authorship with one of the authors JD to the handling editor.
